# Responsible chemistry for a changing world: IUPAC's guiding principles

**DOI:** 10.1039/d5sc08844e

**Published:** 2026-05-01

**Authors:** J. Garcia-Martinez, Peter G. Mahaffy, Mark C. Cesa, Mei-Hung Chiu, Jonathan E. Forman, Mary J. Garson, Richard M. Hartshorn, Tanja Junkers, Leah McEwen, Akiko Nakamura, Daniel O. Reddy, Marvadeen A. Singh-Wilmot, Christine M. Straut Langlinais, Supawan Tantayanon, Rylee E. Van't Land

**Affiliations:** a Molecular Nanotechnology Lab, Department of Inorganic Chemistry, University of Alicante Alicante Spain j.garcia@ua.es; b Chemistry Department, The King's University and King's Centre for Visualization in Science Edmonton Alberta Canada peter.mahaffy@kingsu.ca; c INEOS Nitriles (retired) Wheaton, Ill USA; d Graduate Institute of Science Education, National Taiwan Normal University Taipei Taiwan; e Pacific Northwest National Laboratory Seattle WA USA; f School of Chemistry and Molecular Biosciences, The University of Queensland Brisbane Australia; g School of Physical and Chemical Sciences, University of Canterbury Christchurch New Zealand; h Polymer Reaction Design Group, School of Chemistry, Monash University Clayton Australia; i Cornell University Library Ithaca NY USA; j Colorado State University Fort Collins CO USA; k Department of Chemistry, Queen's University Kingston ON Canada; l Department of Chemistry, The University of the West Indies Mona Kingston Jamaica; m Sandia National Laboratories Albuquerque NM USA; n Department of Chemistry, Faculty of Science, Chulalongkorn University Bangkok Thailand

## Abstract

The practice of chemistry is undergoing rapid transformation driven by innovation, convergence with other disciplines, and an urgent need to address global challenges from climate change to public health. At this critical juncture, the global chemistry community must embrace not only scientific excellence but also a shared commitment to ethical and responsible conduct that is aligned with world needs. Despite the existence of important national and sectoral ethical frameworks, their fragmented application has left a gap in establishing a truly global, inclusive, and actionable reference point for responsible chemistry. In response to this need, the International Union of Pure and Applied Chemistry (IUPAC) has developed and committed to propagating worldwide a set of guiding principles for the responsible practice of chemistry. These principles, developed by chemists for the profession of chemistry, provide a unifying ethical foundation and practical pathways to employ responsible innovation across chemistry research, education, and industry worldwide. These principles aim to provide a common ethical foundation for responsible chemistry worldwide—across sectors, generations, and locations. By aligning scientific excellence with societal well-being and environmental stewardship, these principles offer chemists and institutions tools to foster trust, accountability, and global collaboration.

## Abstract 2

IUPAC has developed a set of guiding principles for the responsible practice of Chemistry worldwide that embrace scientific excellence and nurture a shared commitment to ethical and responsible conduct that is aligned with world needs.

## Graphical abstract

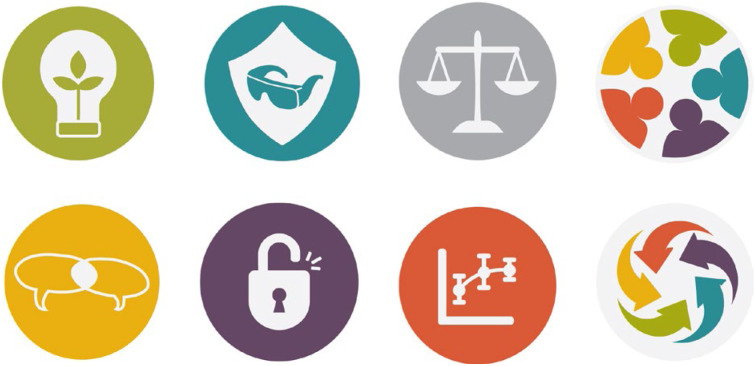

## Introduction

In an age where scientific advancement races ahead at an unprecedented pace, ensuring that chemistry serves the common good without compromising safety, integrity, or sustainability is more vital than ever.^1^ Chemistry touches nearly every aspect of modern life—from healthcare and food production to energy and environmental protection—making its ethical practice not only a professional responsibility but a societal imperative.^2^ To meet these challenges, the global chemistry community has created codes and standards aimed at promoting responsible conduct and preventing misuse of chemical products and processes.

We primarily situate the initiative reported in this manuscript among notable existing guidelines for the responsible practice of chemistry developed from within the chemistry community. These include *The Hague Ethical Guidelines*, facilitated by the Organisation for the Prohibition of Chemical Weapons;^3^ the *Global Chemists’ Code of Ethics*, spearheaded by the American Chemical Society;^4^ the *Chemistry Guide to Ethics*, prepared by the Royal Society of Chemistry;^5^ the *12 Principles of Green Chemistry*, developed by Paul Anastas and John Warner;^6^ the Safe and Sustainable by Design framework for advanced materials and chemicals;^7,8^ the Stockholm Declaration on Chemistry for the Future;^9–11^ and the *Criteria for Sustainable Chemistry*, recently put forward by an international working group.^12^ Each of these initiatives contributes important pieces of a developing shared vision of ethical and responsible chemistry—one that advances human welfare while safeguarding the planet.

However, these initiatives vary widely in scope, focus, and intended audience. In 2015, the working group articulating the Hague Ethical Guidelines collected over 1000 pages of codes of conduct and codes of ethics for the practice of chemistry, demonstrating the long tradition of considering ethical behaviour, but the group concluded that these efforts to date were not very visible to the outside world. Most are either region-specific or emphasize particular aspects of responsible practice (such as green chemistry or chemical security), resulting in a lack of a single globally recognized, comprehensive, and action-oriented framework that can be implemented across educational, academic, industrial, and policy settings. To address this gap, IUPAC has created and committed to propagating the Guiding Principles of Responsible Chemistry, an inclusive, multidisciplinary, and internationally informed framework that complements and extends existing ethical codes while providing a unified foundation for contemporary and emerging chemical practice.

This article introduces the eight IUPAC *Guiding Principles of Responsible Chemistry,* which are intended to foster a culture of responsibility, transparency, and accountability across the global chemistry enterprise. By aligning chemistry research, practice, and education with values that prioritize societal well-being and material and environmental stewardship, the Guiding Principles offer a contemporary framework to support chemists in navigating the complex challenges of our time. In doing so, they invite present and future practitioners of chemistry everywhere to engage not only with what chemistry can do but also what it should do.

Creating a framework of responsibility for chemistry in today's rapidly evolving world presents profound and complex challenges. New technologies—from synthetic biology and nanomaterials to AI-driven chemical discovery—are advancing at breakneck speed, often outpacing our ability to predict their long-term consequences.^13^ At the same time, chemistry is under mounting pressure to help meet the urgent needs of a growing global population, including clean energy, accessible healthcare, food security, and environmental protection. These pressures demand swift and responsible innovation, with deliberate reflection on possible benefits and consequences within the chemistry and broader scientific communities.

Chemistry needs to prioritize working with other disciplines to build trust in science and nurture a healthy science-policy interface to address global challenges in an age characterized by increasing misinformation and global geopolitical tensions. The International Science Council has issued a timely analysis exploring ways to reframe trust in science for multilateral policy.^14^ One key finding is the need for science to both be and appear to be trustworthy. Building trust through responsible, transparent, and inclusive practice is a foundational idea underlying the IUPAC Guiding Principles.

Compounding this complexity is the rich diversity of cultural perspectives, moral traditions, and societal values across regions. Therefore, an effective ethical framework must be flexible and inclusive, capable of guiding responsible scientific conduct while respecting these differences. Such a framework must foster a global culture of accountability and care without imposing a one-size-fits-all model, so that chemistry can truly serve humanity in all its complexity.^15^

Finally, we hope that principles to guide the responsible practice of chemistry will contribute meaningfully to broader conversations about the responsible practice of science, including frameworks such as Science & Technology Studies and Responsible Research and Innovation.

## From principles to actionable guidelines

Traditional ethics frameworks often articulate high-level principles, such as integrity, honesty, and responsibility, which provide essential normative foundations but may not always offer discipline-specific guidance for implementation within particular scientific communities. The new IUPAC Guiding Principles of Responsible Chemistry seek to build upon and put these foundations into practice within the global chemistry enterprise by providing actionable orientation, emphasizing contextual awareness and stakeholder engagement, promoting systems thinking approaches to sustainability, and explicitly integrating ethical reflection into chemistry education.

The Guiding Principles foreground interdisciplinary collaboration, recognizing that ethical challenges in chemistry often intersect with other scientific and societal domains. This encourages chemists to engage with diverse stakeholders and perspectives, enhancing the ethical robustness of their work. The Guiding Principles have also been substantially informed by the synergistic leadership given by other IUPAC projects to champion systems thinking approaches in chemistry education, research, and industrial practice, to ensure that “the solutions to complex global challenges align with ethical considerations, cultural contexts, economic constraints, and societal needs”.^16^ This comprehensive approach fosters a stronger and more practical framework for ethical decision-making in chemistry, addressing the complexities and challenges of modern scientific practice.

As illustrated in the next section, and shown in Fig. 3, each Guiding Principle is accompanied by a structured “Guiding Future Actions” section and a set of discussion questions designed to support putting each Principle into practice in research laboratories, industrial settings, professional societies, and classrooms. These elements provide concrete pathways for embedding the principles into local contexts, including decision-making processes, training programs, innovation pipelines, and institutional reflection mechanisms. By making implementation tools explicit, the initiative seeks to move beyond aspirational statements toward sustained cultural and organizational practice.

## Eight guiding principles of responsible chemistry

The eight IUPAC Guiding Principles were created by chemists for the global chemistry community, with a particular focus on students, national chemical societies, and the professional development of academic and industrial groups. They are intended to have wider utility across the scientific enterprise and industry, and among all those who benefit from the practice of chemistry. The principles were established through a collaborative and iterative process, guided by IUPAC's mission, vision, and core values.^17^ Led by the IUPAC Committee for Ethics, Diversity, Equity, and Inclusion, the initiative “aims to contribute to the worldwide understanding, practice, and application of the chemical sciences, for the betterment of humankind and the environment, present and future”.^18^

The Guiding Principles and accompanying SI were developed by an international group of IUPAC volunteer experts representing multiple regions and professional contexts within the chemical sciences. The composition was not evenly geographically distributed, and the process was primarily situated within the chemistry community, in line roughly with IUPAC's membership distribution. To broaden input, the draft Principles were shared and discussed through international conferences, workshops, webinars, and consultations with committees, professional networks, and external organizations. Feedback from these engagements, spanning academic, educational, policy, and industry contexts, informed successive revisions. The consultation was therefore iterative and multi-modal. While it did not include formal surveys and does not allow precise quantitative accounting by region, it reflects sustained international engagement. Ongoing collaboration with the International Science Council is expanding the range of perspectives involved, and a second IUPAC project is underway to further evaluate and refine the Guiding Principles in light of continued community input.

This collaborative approach mirrors the way IUPAC typically conducts projects, by engaging teams to tackle complex, forward-looking issues that transcend national borders. This process ensured that the Guiding Principles are not only globally relevant but also sensitive to regional nuances and specific challenges in the chemical sciences.

**Fig. 1:**
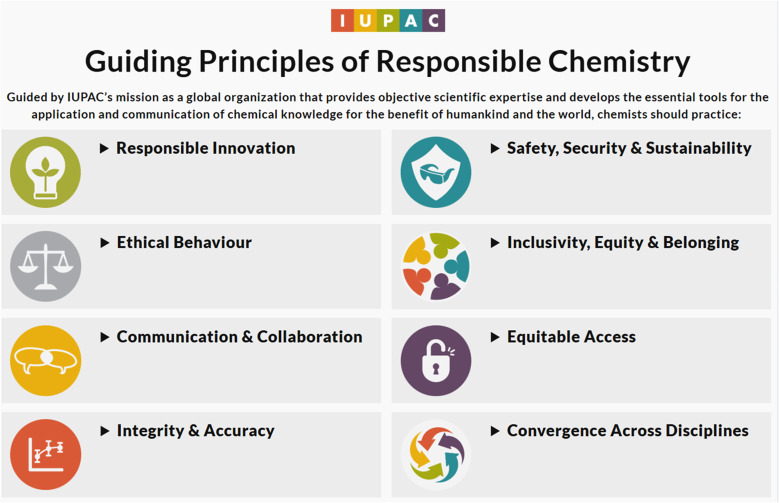
Landing page on the IUPAC website for Guiding Principles of Responsible Chemistry (https://iupac.org/guiding-principles-of-responsible-chemistry/).

The IUPAC Guiding Principles encompass eight key areas, as visualized in Fig. 1, taken from the IUPAC website for the Guiding Principles.

Each of the eight Guiding Principles is summarized with a succinct descriptor, as listed below, along with some examples that show how the Guiding Principles can be applied to the practice of chemistry.

### Responsible innovation

*Employ scientific knowledge and encourage innovations in chemistry to maximize benefits for people and the planet while minimizing and mitigating unintended consequences.* This principle emphasizes that invention and discovery alone are insufficient if their broader impacts, including consideration of possible unintended consequences, are ignored. This principle is grounded in the precautionary approach, systems thinking, and the social contract between science and society. Chemists are uniquely positioned to influence innovative technologies through choices made at the earliest stages of research and development. In practice, this principle calls on researchers and institutions to integrate life cycle thinking, stakeholder engagement, and impact assessments early in the innovation pipeline. It encourages foresight exercises, scenario planning, and multidisciplinary collaboration to anticipate potential risks while maximizing societal and environmental benefits.

In certain circumstances, responsible innovation may also entail a deliberate decision not to pursue, scale, and/or commercialize a line of research when foreseeable harms, inequities, or environmental burdens outweigh anticipated benefits.

### Example of implementation

A multi-partner collaboration in refrigerant design explicitly integrated life cycle analysis at the early stages of discovery to minimize environmental harm.^19^

### Safety, security & sustainability

*Implement a culture of safety, security, sustainability and responsibility in the practice of chemistry.* Without strong safety and security cultures, chemistry cannot sustain public trust or ensure long-term societal benefit. This principle builds on decades of experience in chemical safety, environmental protection, international chemical security agreements, and new emphases on sustainability, and it integrates them under a unified ethos of responsibility. Adopting this principle means embedding safety, security, and sustainability not only as compliance exercises but also as intrinsic scientific values. Approaches to implement this principle include proactive risk assessment, transparent reporting of incidents and near-misses, use of greener and more sustainable chemicals and processes, and international cooperation to prevent misuse of chemicals.

### Example of implementation

Following the Stockholm Declaration on Chemistry for the Future, laboratories may commit to adopt open data platforms to share chemical hazard and sustainability information, accelerating safer design practices.

### Ethical behavior

*Apply ethical values, norms, standards, and judgments to guide the responsible practice of chemistry.* This Guiding Principle anchors all the other principles. It reflects the core idea that chemistry operates within a social context and that the integrity of chemical science depends on individual and institutional choices. This principle draws on established norms such as honesty, fairness, accountability, respect for colleagues, and commitment to the public good. Practical guidance includes ensuring transparent data reporting, acknowledging uncertainty, properly crediting contributions, declaring conflicts of interest, and fostering a climate of integrity in teaching, research, and industrial practice. By operationalizing ethical behavior, chemists strengthen trust within the community and with society at large.

### Example of implementation

A consortium of academic and industrial laboratories established an open reporting protocol during a catalytic materials project. All partners agreed to share raw data transparently, acknowledge uncertainties in their analyses, credit every contributor explicitly, and disclose potential conflicts of interest. This commitment to honesty and accountability fostered trust among collaborators and demonstrated how ethical behavior strengthens both scientific integrity and public confidence.^20^

### Inclusivity, equity & belonging

*Nurture a diverse, equitable, and inclusive global community that incorporates a variety of talents, knowledge, and backgrounds to create a flourishing chemistry enterprise.* Scientific excellence is enhanced by diversity. Drawing on evidence from science policy and organizational studies, inclusive environments lead to better problem solving, more innovative ideas, and more just outcomes. In chemistry, a global discipline with diverse applications, ensuring equitable participation is both an ethical obligation and a scientific imperative. Practical actions include creating accessible career pathways, ensuring fair access to resources, removing structural barriers, amplifying marginalized voices, and embedding inclusivity into the design of research teams, curricula, and conferences.

### Example of implementation

A regional university alliance in the Caribbean used these principles to co-develop inclusive research training programs with underrepresented groups.^21^

### Communication & collaboration

*Communicate knowledge and practices through education and outreach to equip chemists and the public with the necessary understanding, tools, and values to benefit people and the planet.* Clear, transparent, and responsible communication is essential for chemistry to maintain societal legitimacy and foster interdisciplinary collaboration. This principle is grounded in the notion of shared responsibility between scientists and society. In practice, it involves promoting open science, sharing data responsibly, engaging non-specialists, supporting science education, and ensuring that communication and collaborations are accessible across languages and cultures. This principle also calls on chemists to collaborate across disciplines and sectors to address complex global challenges.

### Example of implementation

Through “*The Global Conversation on Sustainability*,” IUPAC and the International Younger Chemists Network (IYCN) connect universities, chemical societies, and industry partners worldwide to organize coordinated events and dialogues on sustainability. This initiative, held every 25 September, fosters open communication, shared learning, and collaboration across sectors and regions, enabling the global chemistry community to align actions toward common sustainability goals.^22^

### Equitable access

*Provide equitable access to information, resources, and opportunities to create an open, inclusive, and collegial environment for the chemistry community.* This principle reflects the recognition that the benefits of chemistry should not be limited to a few countries, institutions or specific population groups. Equitable access underpins scientific justice; it allows ideas, talents, and technologies to flourish globally. It aligns with open science movements and global capacity-building initiatives. Practical actions include making research outputs open and interoperable, sharing data and tools, fostering equitable collaborations across regions and contexts, and ensuring that capacity-building is sustainable rather than extractive.

### Examples of implementation

IUPAC has supported capacity-building initiatives, such as the Safety Training Program, which provided scientists from underrepresented regions with access to shared resources, training opportunities, and international research collaborations.^23^

### Integrity & accuracy

*Use and interpret data, models, and theories with integrity, completeness, and accuracy, and make use of the latest technological innovations ethically, responsibly, and fairly.* These core values are the backbone of credible science. This principle reaffirms chemists' responsibility to ensure that knowledge generation and use are trustworthy. With the rise of automated data generation and AI-driven modeling, the need for rigorous validation, transparency, and reproducibility has never been greater. Chemists are called to adopt open data standards, rigorous documentation, error reporting, and careful interpretation of uncertainty. We must also apply critical judgment when adopting new technologies to avoid unintended misuse or bias.

### Example of implementation

Through the WorldFAIR Project, IUPAC, CODATA and partners develop interoperability frameworks and FAIR data guidelines for chemistry; researchers assign persistent identifiers and rich metadata to datasets and deposit them into trusted repositories, making data findable, accessible, interoperable and reusable and strengthening integrity and reproducibility.^24^

### Convergence across disciplines

*Promote the convergence of chemistry with other disciplines to address global issues and ensure ethical development and the well-being of people and the planet.* This principle reflects the growing reality that the most pressing challenges—climate change, energy transition, sustainable materials, health and security—cannot be solved by chemistry alone. Convergence fosters holistic problem solving, ethical co-design, and integration of diverse ways of knowing. Practically, it means forging partnerships with engineers, data scientists, policymakers, social scientists, the humanities, artists, and communities. This principle emphasizes shared responsibility, interdisciplinary literacy, and co-creation of solutions.

### Example of implementation

The Science Missions for Sustainability initiative, led by the International Science Council, mobilizes interdisciplinary teams by bringing together chemists, engineers, climate scientists, and social scientists to co-design and implement solutions to global challenges such as decarbonization, clean water, and food security.^25^

The IUPAC Guiding Principles focus on advancing chemistry globally through collaboration across disciplines, as well as research integrity, transparency, and inclusivity. They build on and envelop other codes and guidelines mentioned earlier, which address either specific aspects of the practice of chemistry or specific national or geographical audiences.

With their broad scope, which encompasses all aspects of chemistry and IUPAC's global reach, IUPAC's Guiding Principles extend beyond technical sustainability to foster responsible innovation, equitable access to information, and enriched practice through a culture of diversity within the scientific community. By promoting both personal responsibility and collective global action, the IUPAC Guiding Principles invite the chemistry community to adopt a unifying standard, ensuring that the practice of chemistry is guided by a commitment to be both responsible and trustworthy. This commitment is crucial for advancing ethical, inclusive, and sustainable science that benefits society and the environment. By aligning with international standards and promoting ethical conduct, IUPAC reinforces the importance of chemistry in addressing global challenges responsibly.

## Engaging with the guiding principles

**Fig. 2:**
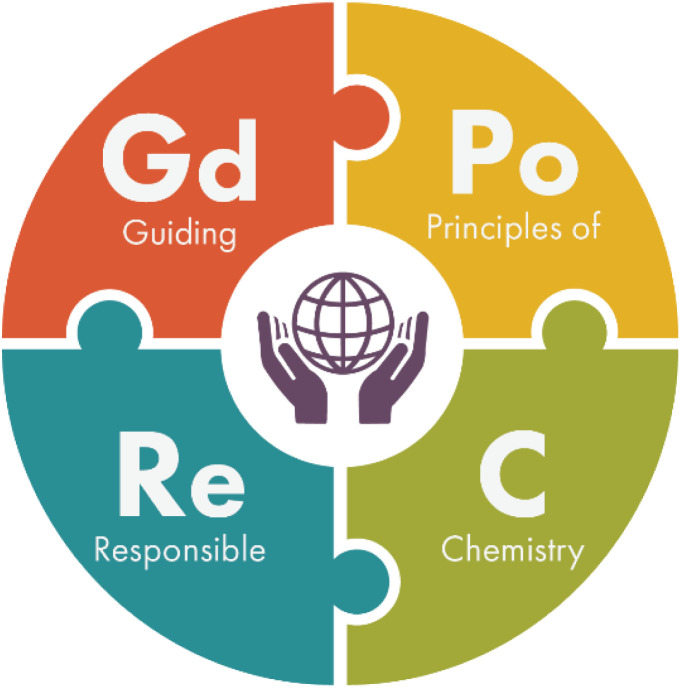
Icon on the IUPAC home page directing users to the new site IUPAC Guiding Principles for Responsible Chemistry.

**Fig. 3:**
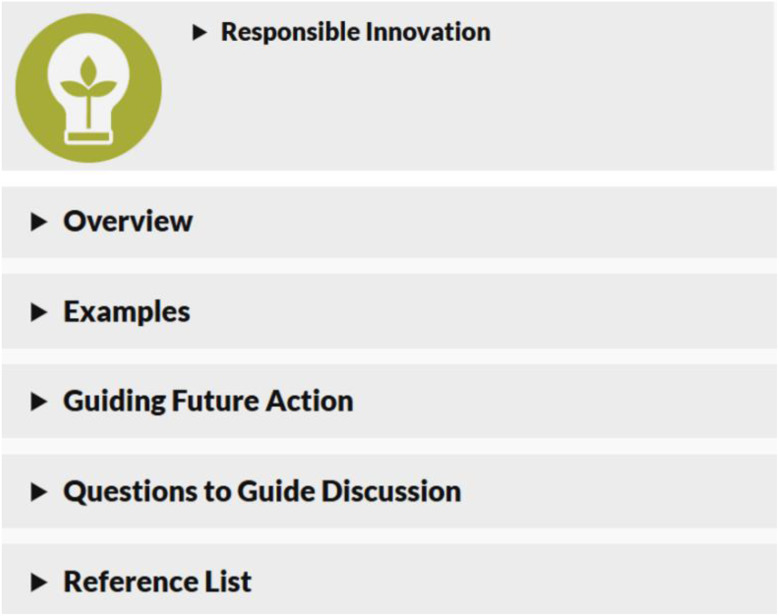
Dropdown menu from the Responsible Innovation Principle, showing the sections described below.

The Guiding Principles are profiled on IUPAC's home page, with an icon (Fig. 2) directing viewers to the Guiding Principles website (https://iupac.org/responsible-chemistry/). On the IUPAC website, the home page shown in Fig. 1 opens, and clicking on a Principle opens a drop-down menu (Fig. 3 shows the “*Responsible Innovation”* Guiding Principle) with (a) an overview of the Principle, (b) examples, (c) some ways to guide future action pertinent to the Principle, (d) discussion questions to be used by individuals or groups, such as university classes or professional development sessions, and (e) key references.

Feedback from university science students piloting the Guiding Principles highlighted the value of engaging with specific examples and future actions that encourage a deeper understanding of the real-world implications of scientific advancements. With that in mind, each section in the drop-down menu for each Guiding Principle (Fig. 3) delves deeper into the topic, as illustrated below.

### Examples section for each principle

Following a brief overview, each Guiding Principle introduces practical, real-world examples that demonstrate the benefits and unintended consequences of the practice of chemistry. Some examples embedded in the website include:

*“Responsible Innovation”* features three examples: the Leblanc process, which transformed industry but also led to environmental damage; the Haber-Bosch process, whose benefits for agriculture come with significant ecological and energy costs; and past and present innovations to develop refrigerants, which demonstrate the importance of life cycle analysis and systems thinking to avoid unintended consequences. These examples illustrate the “two faces of chemistry,” demonstrating the complexities of innovation and emphasizing the importance of anticipating potential risks, unintended consequences, and environmental and social impacts.

*“Communication & Collaboration”* includes an example taken from The Muscat Declaration on Global Science, published by the International Science Council in January 2025. This initiative brought together over 250 international organizations from diverse cultural, disciplinary, and institutional backgrounds to demonstrate how open, inclusive communication can bridge values and perspectives to produce shared commitments.^26^

### Guiding Future Action section for each principle

Suggestions for implementing the interconnected ideals of each principle offer concrete ways to adopt, implement, and promote the Principles of Responsible Chemistry. Some examples on the website include:

*“Safety, Security & Sustainability”* provides five key points and outlines specific actions derived from the Stockholm Declaration on Chemistry for the Future (https://www.stockholm-declaration.org/), published in May 2025 after a convening of leading global experts in green and sustainable chemistry. The Declaration calls upon all chemists to design chemicals and processes that prioritize safety, promote open access data and information relating to the safety and sustainability of each chemical, and advocate for policies that disincentivize polluting, toxic, and dangerous chemical practices.

*“Inclusivity, Equity & Belonging”* offers actions to address the persistent challenges that hinder progress. These suggestions include a focus on institutional commitment to diversity and inclusivity, continuing professional development, and incorporating examples of inclusivity into chemistry education.

*“Equitable Access”* explores concrete strategies for how the chemical and wider scientific communities can work to eliminate barriers to equitable access in educational and academic settings. These strategies include increasing the availability of open access teaching and research resources, sharing infrastructure and data, systematic training, improving facilities for disabled students and scientists, and developing explicit strategies or policy frameworks built around inclusivity.

*“Convergence Across Disciplines”* articulates steps that can be taken to support interdisciplinary convergence, such as pursuing collaboration across a diverse range of fields and developing curricula that highlight chemistry connections to other disciplines through systems thinking.

### Questions to Guide Discussion section for each principle

**Fig. 4:**
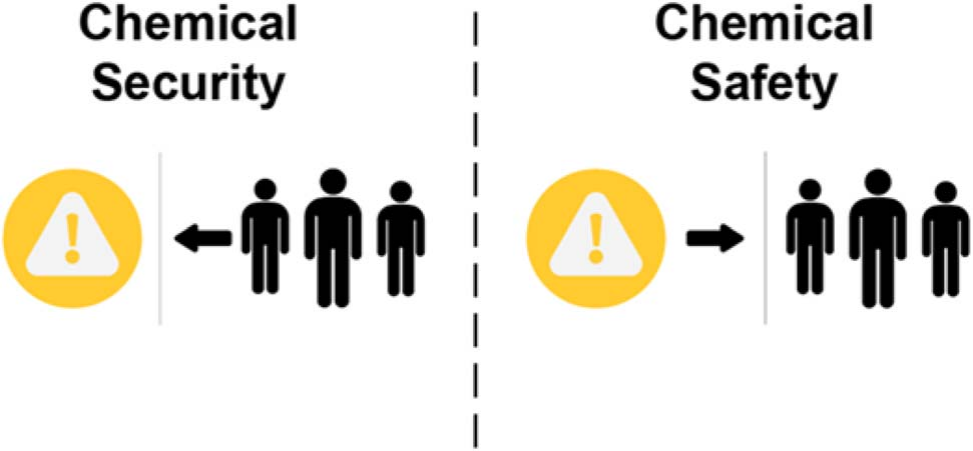
Distinguishing between chemical security (protecting chemicals from harmful uses by people) and chemical safety (protecting people and the environment from hazardous materials).

Critical thinking, self-reflection, and engagement with each Guiding Principle are encouraged through questions that challenge users to explore how responsible chemistry can be embedded in research, practice, and education from the start. Some examples on the website include:

*“Safety, Security & Sustainability”* includes questions that build understanding of the differences between the concepts of safety and security (Fig. 4) and invites readers to give examples from their experience where sustainability practices enhanced chemical safety and security in a workplace, laboratory, or community.

*“Communication & Collaboration”* invites readers to refer to other Guiding Principles, such as *“Responsible Innovation”* or *“Convergence Across Disciplines”* and identify an example where greater collaboration between chemists and other professionals might have led to fewer unintended consequences.

*“Integrity & Accuracy”* asks readers to think about how computers and software programs “know” how to interpret data, and how much scientists rely on context to interpret a data set.

### References section for each principle

For users wishing to further explore key ideas, a curated set of references is provided at the end of each Guiding Principle.

## Global collaboration and implementation

A strength of this IUPAC initiative is that it has brought together a diverse group of chemists to develop a framework for the responsible practice of chemistry. We believe that change in the chemistry domain primarily needs to start from within. On the other hand, this also serves as a limitation as other perspectives including those of experts in the social sciences have not yet been directly involved in creating this framework. Principle-based guidelines for practice are known to have potential for further limitations including detachment from local contexts and their site of implementation, creating a principle-to-practice gap, and the potential for detachment from cultural, social, political, and economic contexts from which they are used.^27^

To mitigate these limitations, the “Examples”, “Guiding Future Actions", and “Questions for Discussion” sections of the Principles encourage application by local communities to diverse contexts in which they will be implemented. Development of the Principles also was enriched by reviews involving multiple IUPAC committees and task forces. IUPAC's Committee on Ethics, Diversity, Equity, and Inclusion plays a pivotal role in securing funding and coordinating efforts to promote these principles globally. By engaging with diverse stakeholders, including educators, industry professionals, policymakers, and young people, IUPAC ensures that the principles are relevant and adaptable to various cultural and institutional contexts.

At the opening of the IUPAC World Chemistry Congress and General Assembly in Kuala Lumpur, 12–19 July 2025, the Guiding Principles initiative was officially launched along with the dedicated IUPAC website. The Guiding Principles are not intended to be a one-time, static initiative. Rather, the chemistry community is encouraged to help IUPAC ensure that the Principles and their SI remain up to date by engaging with groups who will use them to catalyze discussion. Examples of the diverse contexts for propagation and feedback include sessions at the World Chemistry Congress, The International Chemical Conference of Pacific Basin Societies, the Atlantic Basin Conference on Chemistry, the European Chemical Society, the African Conference on Research in Chemistry Education, an international webinar hosted by the Royal Society of Chemistry (UK), and national conferences in the United States, Canada, and India. These forums invite experts and stakeholders from diverse international contexts to share insights, address challenges, and engage in meaningful dialogue about how to integrate these principles into everyday chemical practices. These initiatives mark significant steps toward promoting responsible innovations in chemistry on a global scale.

The Guiding Principles and educational materials will be translated into the official languages of the United Nations in the second phase of the IUPAC project to ensure equitable global dissemination and use. Mapping of the Guiding Principles onto secondary and post-secondary chemistry curriculum has begun, starting with the International Baccalaureate curriculum.

We encourage other disciplines and interdisciplinary groups to explore similar initiatives aimed at articulating responsible practices within their fields. IUPAC actively invites collaboration on these endeavors, recognizing that diverse perspectives are essential for fostering a comprehensive understanding of responsible innovation. By engaging with a wide range of stakeholders, we can collectively work towards minimizing bias and ensuring that ethical considerations are inclusive and reflective of the broader societal context.

In this spirit, the Guiding Principles are conceived as a living document. Now that they have been formally published, they are intended to be continuously enriched through broad participation from the global chemistry community and other stakeholders. This article represents an additional avenue for propagating the Guiding Principles, encouraging engagement, and inviting constructive input to further refine, contextualize, and strengthen them over time.

## Conclusions

The IUPAC Guiding Principles of Responsible Chemistry are intended as a significant advancement in promoting standards for ethical and responsible practice within the chemical sciences. They are distinctive in offering a globally inclusive, action-oriented, and interdisciplinary framework that is adaptable across geographies and institutional contexts. The principles emerge as a starting point for the responsible practice of chemistry, championed by IUPAC, which sets global standards for the chemistry community. IUPAC is explicitly open to enrichment and elaboration of these principles by others as the Guiding Principles are refined and propagated. Through comprehensive educational initiatives and global collaboration, these principles are poised to profoundly influence future generations of chemists and to build synergy with other initiatives, such as a new emphasis on the making of virtuous chemists.^28^ In this sense, the principles support the cultivation of chemists who combine technical excellence with integrity, humility, and a commitment to serving the public good, ensuring that scientific expertise is exercised with ethical awareness and civic purpose. By integrating these guidelines into educational frameworks and professional practices, the chemistry community can ensure that scientific progress aligns with societal values and contributes positively to global well-being.

We invite everyone interested in these efforts to learn more and access the full set of principles and supporting resources at our website,^29^ where educational materials, implementation guidance, and opportunities for engagement are continuously updated. We are actively soliciting feedback from a broad group of stakeholders and look forward to capturing and incorporating suggestions over the next year leading to an updated release of the Guiding Principles.

As chemistry continues to evolve and intersect with various disciplines, a commitment to ethical and responsible practices will remain paramount. IUPAC's efforts to develop and promote these guiding principles exemplify the proactive steps necessary to uphold the integrity and societal relevance of the chemical sciences and to nurture trust in science among the publics we serve through chemistry.

## Author contributions

JGM (conceptualization) brought the vision for this initiative to IUPAC. JGM and PGM (writing – original draft) drafted and edited this manuscript. MCC and MJG (project administration) co-chaired the IUPAC project task group that envisioned, created, and edited the Guiding Principles. RVL (visualization) was the lead on creating icons and figures for the Guiding Principles and edited this manuscript. All authors (formal analysis and methodology) contributed to drafting and editing the Guiding Principles and some authors offered edits to the manuscript (writing – review & editing).

## Conflicts of interest

There are no conflicts to declare.

## Data Availability

No new data were generated in this perspective.
